# Numerical Investigation on the Influence of Turbine Rotor Parameters on the Eddy Current Sensor for the Dynamic Blade Tip Clearance Measurement

**DOI:** 10.3390/s24185938

**Published:** 2024-09-13

**Authors:** Lingqiang Zhao, Fulin Liu, Yaguo Lyu, Zhenxia Liu, Ziyu Zhao

**Affiliations:** 1 School of Power and Energy, Northwestern Polytechnical University, Youyi West Road 127#, Xi’an 710054, China; lqznpu@mail.nwpu.edu.cn (L.Z.); fulinliu@mail.nwpu.edu.cn (F.L.); zxliu@nwpu.edu.cn (Z.L.); 2 Xi’an Research Institute of High-Tech, Xi’an 710054, China; zziyu@mail.nwpu.edu.cn

**Keywords:** eddy current sensor, dynamic blade tip clearance, finite element model, calibration, rotor parameters

## Abstract

Eddy current sensors are increasingly being used to measure the dynamic blade tip clearance in turbines due to their robust anti-interference capabilities and non-contact measurement advantages. However, the current research primarily focuses on enhancing the performance of eddy current sensors themselves, with few studies investigating the influence of turbine rotor parameters on the measurements taken by these sensors for dynamic blade tip clearance. Hence, this paper addresses this gap by using COMSOL Multiphysics 6.2 software to establish a finite model with circuit interfaces. Additionally, the model’s validity was verified through experiments. This model is used to simulate the voltage output of the sensor and the measurement of dynamic blade tip clearance under various rotor parameters. The results indicate that the length and number of blades, as well as the hub radius, significantly affect the sensor voltage output in comparison to rotation speed. Furthermore, we show that traditional static calibration methods are inadequate for measuring dynamic blade tip clearance using eddy current sensors. Instead, it is demonstrated that incorporating rotor parameters into the calibration of eddy current sensors can enhance the accuracy of dynamic blade tip clearance measurements.

## 1. Introduction

The efficiency and performance of rotating machinery are greatly influenced by the blade tip clearance (BTC), which is the distance between the blade tip and the casing. Excessive clearance can lead to a sharp loss of power. For example, an increase in blade tip clearance by 1 percent of the turbine blade length may cause 1 to 3 percent efficiency loss in the turbine stage in aircraft engines [1,2]. On the other hand, the small tip clearance design can result in collision and friction between blades and case, leading to engine surge and potential accidents, reducing the safety margin of the equipment [3,4]. Therefore, it is particularly important for maintaining and improving the performance of rotating machinery and providing key information for the active control of aircraft engines by a real-time (BTC) system.

In the past few decades, various gap measurement sensors based on different measurement principles have been developed and applied, such as capacitive, fiber optic, microwave, probe, and eddy current sensors. Among them, capacitive sensors are widely used for BTC due to their simple structure and low cost. However, the output parameters of capacitive sensors are not only affected by the gap changes but also by the humidity and pressure of the gas between the capacitor plates, posing challenges for their application in turbine blades of aircraft engines [5]. Fiber optic sensors offer high measurement accuracy, small probe size, and are not limited by the structure or material of the blade, but their probe surface is sensitive to carbon black, oil residue, and dust, limiting their use to cleaner areas of aircraft engines such as the fan and compressor blades [6,7]. Although microwave sensors have characteristics like high-temperature resistance and resistance to gas corrosion, the spatial filtering effect limits their application [8]. Despite their advantages of simple working principles, high-temperature tolerance, and independence from the shape of the adjacent blade end faces, probe sensors are limited by their ability to measure only the smallest tip clearance among all blades so that probe sensors are gradually being phased out from tip clearance measurement and application [9]. Eddy current sensors (ECSs) have advantages such as non-contact operation, high-temperature resistance, good reliability, fast response time, robust anti-interference capabilities, and the ability to work in harsh environments. In recent years, it has been widely researched and applied in the measurement of BTC in the turbine section of aircraft engines [10,11,12].

The research on eddy current sensors for BTC has made significant progress in recent years. From 2013 to 2018, researchers from Oxford University, led by K.S. Chana, developed eddy current sensors capable of withstanding temperatures exceeding 1500 K. Additionally, performance tests were conducted on the RB168Mk 101 Spey engine and Rolls-Royce VIPER engine [13,14,15]. The test results not only show that the ECS is feasible for measuring turbine BTC but also that the results are reliable. Additionally, based on eddy current technology, K.S. Chana and V. Sridhar developed the blade tip timing system (BTT), which utilizes optimized algorithms to build simple, reliable, real-time, and cost-effective analog electronic circuits [16]. This has promoted the application of the system in health monitoring for aircraft engines. Jiang Zhe et al. have simplified the calibration process of eddy current sensors by introducing new mutual inductance calculation methods [17,18]. Based on the structural characteristics of blade tip clearance in rotating machinery, Tianli Hu et al. established blade array models to analyze the spatial filtering effects in blade tip clearance measurements. In addition [19], Zhao Z.Z et al. [20,21,22] focused on the high temperature ECS design and validation. The simulation and experiment method were both used to determine the optimal sensor structure parameters and materials. Nidhal Jamia et al. conducted an in-depth study on the effects of blade geometry, and rotation velocity on voltage output of the passive ECS in blade tip timing (BTT) by building quasi-static finite element analysis models. Furthermore, a test rig was designed and manufactured to validate the result [23,24].

In recent years, researchers have neglected the influence of blade length, blade number, the rotor radius, sensor calibration, and their relative velocity on the accuracy of blade tip clearance measurement. With the increasing demand for measurement accuracy in BTT systems, the high cost and extensive resources required for experiments make it challenging to rely solely on experimental methods to optimize measurement systems. Therefore, establishing a finite model including both the ECS and the rotating bladed disk for optimizing sensor system design is required.

To overcome the above limitations, the innovation of this article lies in establishing a 2D finite element model to simulate the integrated measured output by using electromagnetic fields, moving mesh ports and circuits in COMSOL Multiphysics software. Then, this model is employed to simulate the effects of rotor rotation speed, number of blades, blade length, and hub radius on the output parameters of ECS. Furthermore, the study evaluates the impact of these factors on measurement results when a static calibrated sensor is used for dynamic blade tip clearance (BTC) measurement.

## 2. Principle of ECS in BTC Measurement

**Figure 1:**
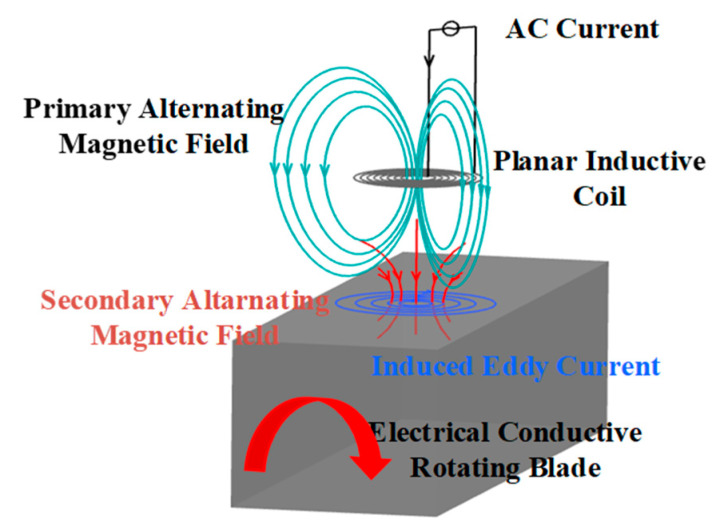
Schematic diagram of measuring principle.

The fundamentals of eddy current gap measurement are Faraday’s law of electromagnetic induction and Lenz’s law. The ECS is excited by a high-frequency AC signal and then generates a primary alternating magnetic field. When a moving metallic blade passes through the magnetic field, an eddy current is induced in the blade. Then, the induced current in the blade generates a secondary alternating magnetic field that is contrary to the primary alternating magnetic field, as shown in Figure 1. Finally, the change in parameters of the coil is captured by the ECS.

**Figure 2:**
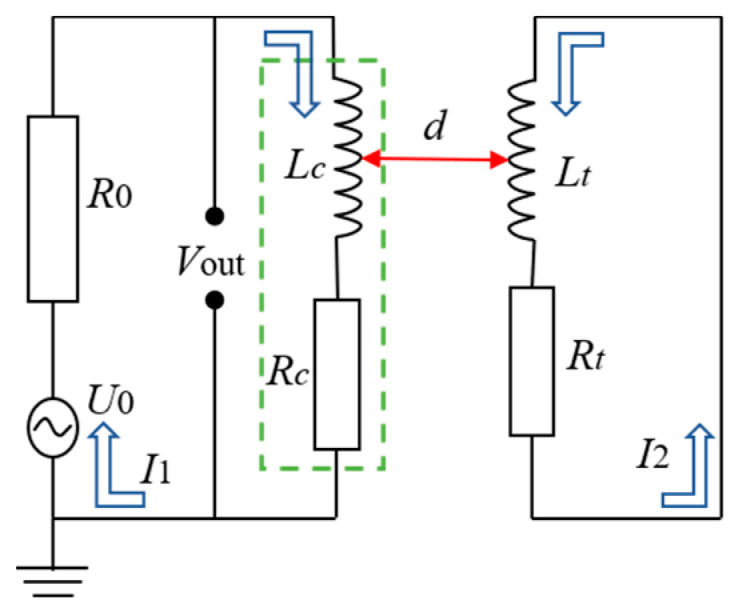
Equivalent circuit of measurement.

Figure 2 shows the equivalent circuit diagram of the measurement. The change in parameters of the coil is related to the degree of magnetic coupling between the blades and the coil. In other words, the clearance between the sensor and the blade changes can be monitored by detecting the coil voltage output (*V_out_*). Therefore, the ECS can be applied to the BTC system by establishing the relationship between sensor output voltage and distance between the blades and the coil.

## 3. Numerical Modeling

### 3.1. Finite Element Analysis Model

**Figure 3:**
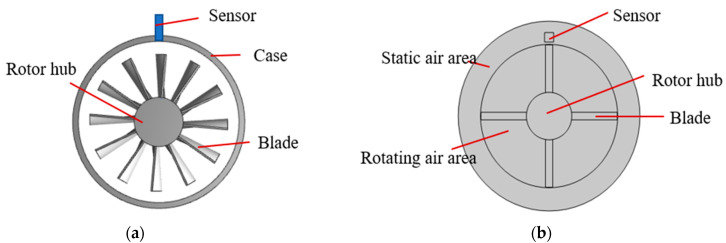
The model for BTC measurement: (**a**) Practical model for BTC measurement; (**b**) 2D simplified model.

Here, the FEA is adopted, and the practical model is simplified as a two-dimensional model to save the calculation time. The simplified geometry model is shown in Figure 3. The geometry is cut along the air gap into two adjacent parts: one static part of the model, which consists of the sensor and static air area, and the moving part, composed of the disk and rotating air area, as shown in Figure 3b. The moving and static regions are then coupled using the ‘Form Assembly’ option in COMSOL, which allows a controlled continuity in the scalar magnetic potential at the interface. Then, we set the moving part to the rotation domain through the dynamic mesh interface in the physical field of rotating machinery, and the magnetic, which can directly convert the moving part into the rotating domain, and the rotor speed are set in the rotating domain interface. The continuity of consistent boundary pairs in the physical field must be set at the continuity interface to maintain the continuity of the magnetic scalar potential at the interface between the rotating and static parts.

The governing equations for electromagnetic field FEA are Maxwell’s equations, and their differential forms are shown as Equation (1).

(1) $$\left.\begin{array}{c} \nabla \cdot H=J\cdot \frac{\partial D}{\partial t}\\ \nabla \times E=-\frac{\partial B}{\partial t}\\ \nabla \cdot D=\rho \;\;\;\;\;\;\;\;\;\\ \nabla \cdot B=0\;\;\;\;\;\;\;\;\; \end{array}\right\}$$

where *H*—magnetic intensity vector, A/m; *J*—ampere density vector, A/m^2^; *D*—electric displacement vector, C/m^2^; *E*—electric field intensity vector, V/m; *B*—magnetic induction intensity, 1T = 1Wb/m^2^; and *ρ*—electric charge density.

Based on the magnetic vector potential and the magnetic scalar potential, the COMSOL Multiphysics software solves the Maxwell’s equation well. It can be modeled with the Ampere’s law feature in the software. Furthermore, the magnetic scalar introduces fewer degrees of freedom and ensures better accuracy of the magnetic flux density coupling when it is used to enforce the continuity of both the static part and the moving part. The eddy current sensor is modeled by using the COMSOL interface called coil domain, which models a conductive domain subject to a lumped excitation, such as voltage, current, or circuit. The circuit interface can be added through the physical field in the software.

**Figure 4:**
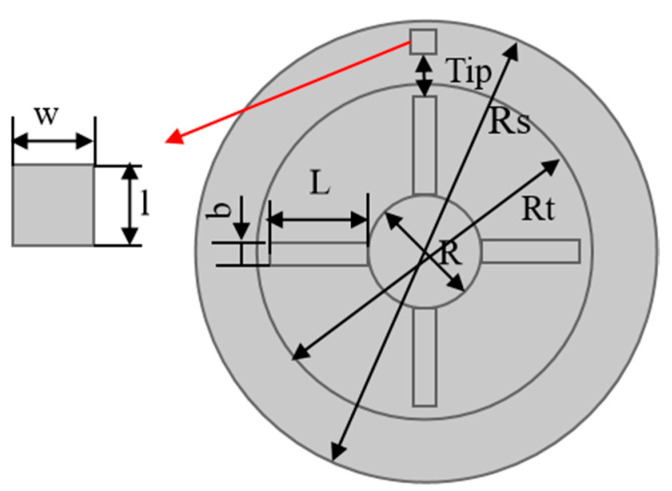
The model geometry.

Therefore, combining the equivalent circuit of measurement as shown in Figure 2 and the built-in circuit interface in the software, we establish the calculation model; the components, including grounding, voltage source, resistor, and outer I vs. U, were added through the circuit interface. Additionally, we apply excitation signals (voltage and frequency) with the COMSOL Multiphysics software in the voltage source component. The sensor made of the spiral coil without the magnetic core is simplified to a rectangle, as shown in Figure 3b. The computational domain contains air, sensor and blade, and rotor hub. The sensor coil is made of platinum, and the blade and rotor hub are made of aluminum. The design parameters are shown in Table 1 and Figure 4.

### 3.2. Data Processing

**Figure 5:**
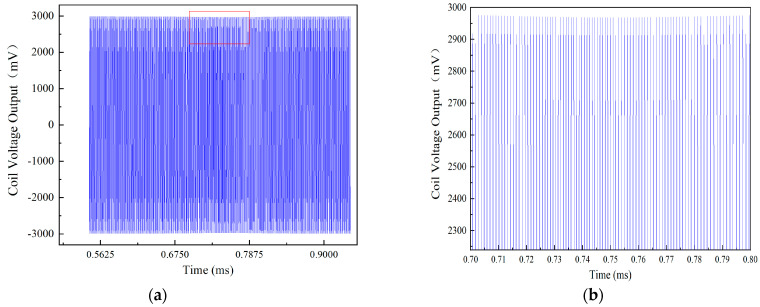
The output signal waveform at a rotation speed of 20,000 r/min: (**a**) the raw signal; (**b**) the partially enlarged image of the figure (**a**).

**Figure 6:**
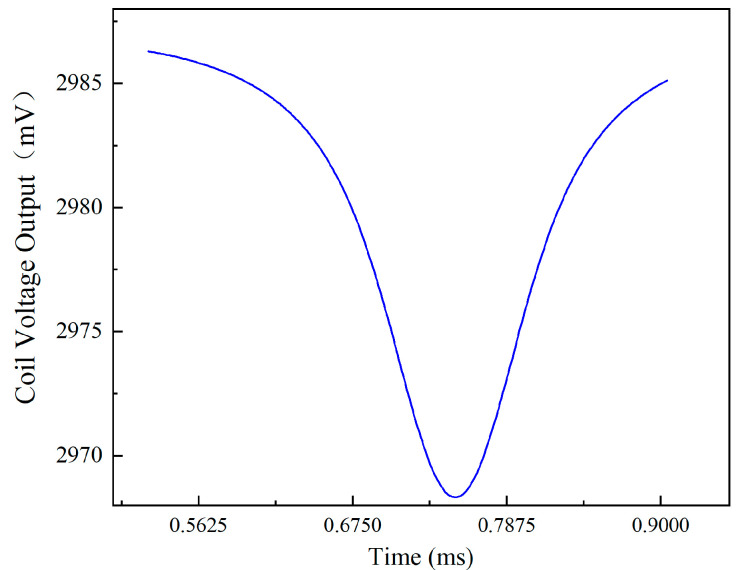
The processed signal.

The raw voltage signals of the coil obtained from the COMSOL Multiphysics software are relatively complex and do not intuitively reflect the relationship between blade rotation and sensor output, as illustrated in Figure 5. The example shows a rotation speed of 20,000 r/min in the figure. To accurately represent the voltage variation of the coil caused by the moving blades in the turbine, the raw signal is processed using a band-pass filter and peak value extraction via MATLAB. The processed signal is shown in Figure 6. At t = 0.75 ms, one of the blades passes the ECS, causing the sensor voltage output to reach a valley, as shown in Figure 6. When the blade moves away from the sensor, the voltage output decreases; otherwise, it increases. This means that the valley of the sensor voltage output represents the blade tip clearance.

### 3.3. Mesh Refinement Study

**Figure 7:**
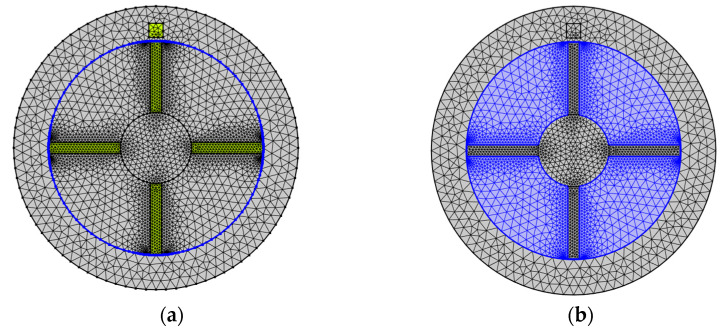
Mesh of the calculation domain: (**a**) rotating boundary; (**b**) the rotating air domain.

In terms of meshing, triangular elements were used, as shown in Figure 7a. Considering the skin effect and the highest eddy current density in the blade and coil, a refined mesh is applied at the coil and blade surfaces to improve calculation accuracy, highlighted in yellow in Figure 7a. Additionally, due to the creation of an assembly, the static part and rotating part are meshed separately, which is evident from the distinct positions of the mesh nodes on each side of the interface in Figure 7a. A fine mesh is applied on the continuity boundary, highlighted in blue in Figure 7a. In particular, the rotating part of the boundary is meshed more finely than the stationary part to ensure accurate continuity at this boundary, thereby enhancing model stability, as highlighted in blue in Figure 7b. Due to COMSOL’s support for the moving mesh approach, these two parts with their corresponding meshes remain in contact at the boundary.

**Figure 8:**
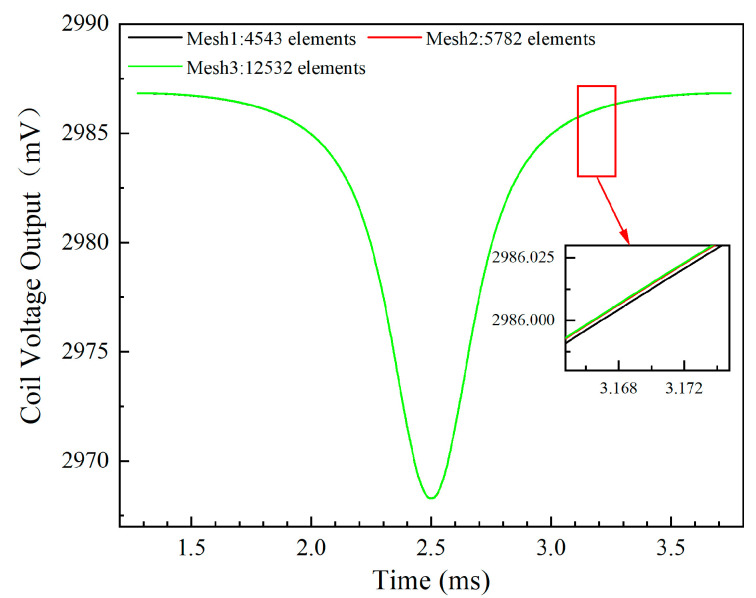
Effect of mesh refinement on the sensor voltage output corresponding to different meshes.

Given the variation in mesh density across different domains within the model’s geometry, it is essential to perform a mesh refinement study to ensure convergence of the results. Utilizing the mesh processing capabilities built into COMSOL, three different levels of mesh refinement were employed: refine, finer, and ultrafine. Additionally, adaptive mesh refinement was added in the transient solution setup window, which provides more accurate computational results. The influence of mesh density on the coil voltage output is depicted in Figure 8. As the number of cells increases, the output voltage tends to stabilize. Furthermore, the enlarged view in Figure 8 indicates that the impact of mesh density on the computational results becomes negligible when the number of cells reaches 12,532. Consequently, the subsequent research presented in this article utilizes the ‘ultrafine’ mesh refinement method for further analysis.

### 3.4. Setting the Time Step

For most transient wave problems, the built-in transient solver continuously adjusts the time step by default to meet the tolerance specified in COMSOL. The relative tolerance box in the ‘Transient’ settings controls how the solver adjusts the time step. The smaller this value, the smaller the time step, and the higher the accuracy. Note that changing the number of output time steps in the transient node only controls the output time steps and has little effect on the actual time steps used by the solver. The relative tolerance is 0.0001, and the output data frequency (sampling frequency) should be 20 times the excitation frequency based on the Nyquist theorem. Therefore, the time step is t in Table 1. The model is then simulated in the time domain for over one complete rotation.

### 3.5. Numerical Computation Model Validation

**Figure 9:**
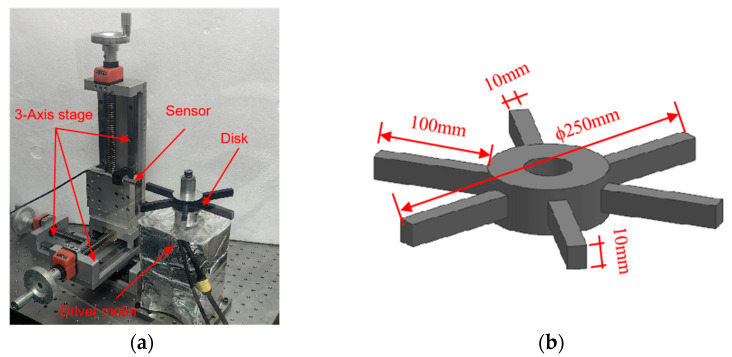
(**a**) Illustration of testing setup; (**b**) image of simplified tested disc made of aluminum.

To verify the numerical computation model, the testing setup was designed and manufactured as shown in Figure 9. The pre-balanced bladed disk with 6 blades was mounted horizontally and was connected to the driver motor (80BL01 220V brushless motor, made by Okoda stepper motor in China) via a stainless steel shaft. The rotational speed could be regulated from 100 r/min to 18,000 r/min, measured by an optical tachometer (LG9200 made by Xiaoye Measurement Technology). The sensor was secured by a fixture attached to the 3-axis high-precision stage. Additionally, the clearance between the sensor and blade was controlled by the 3-axis high-precision stage, the Z-axis adjustment precision is 0.5 μm. Additionally, a sensor coil was manufactured with the same parameters as the calculated coil, resulting in an inductance value of 12.54 μH. Moreover, after six repetitive experimental validations, the uncertainty in the sensor’s output peak voltage at 3mm was 0.063%.

**Figure 10:**
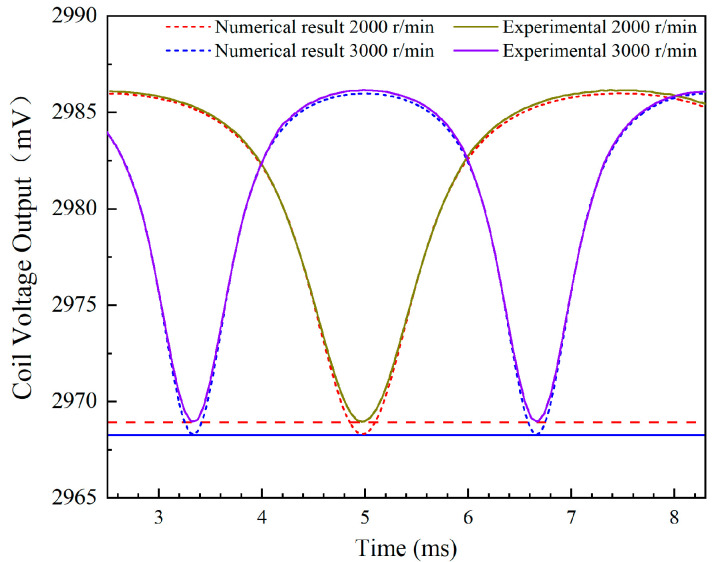
Comparison of the numerical calculations with the experimental results.

Prior to conducting the tests, the test system was subjected to five repetitive trials. The test results indicated that the relative uncertainty of the test system was 0.528%. These findings demonstrate that the measurement system exhibits notably high repeatability and accuracy. Then, based on the principles of centrifugal force and material mechanics formulas, the maximum elongation of the blade at the rotation speeds of 2000 r/min and 3000 r/min were calculated to be 0.541 μm and 0.912 μm, respectively. This elongation is sufficiently small to be negligible, thereby having no significant impact on the measurement results. Therefore, tests with 2000 r/min and 3000 r/min were conducted, and the gap of 3mm between the sensor and the blade tip was adjusted. The coil voltage outputs were collected and compared to the simulated data from the described models as Figure 10. The minimum value indicates the moment when the blade is directly aligned with the sensor, and the mean absolute error between the calculated and experimental voltage values was found to be 0.8mV. Furthermore, the patterns observed in the numerical calculations and experiments are consistent when the blade passes through the sensor. The comparison results indicate that the model developed in this study is reasonable and reliable.

## 4. Results and Discussion

### 4.1. Characteristic Calibration

The common static calibration method of ECS for blade tip clearance measurement, as described in the literature [18], involves selecting a target with the same end face shape and material as the measured blade and placing it on a movable platform. After properly adjusting the equipment, the target is moved at a certain speed while adjusting the distance between the target and the sensor. The sensor output is then recorded to obtain the calibration curve.

**Figure 11:**
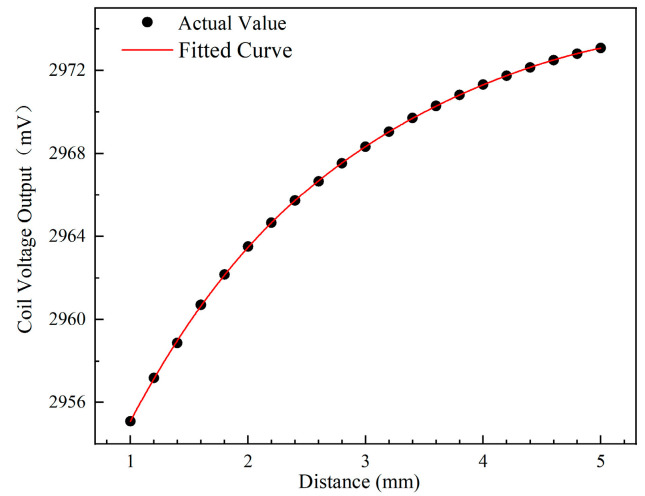
Calibration curve.

Following this method, the sensor in the model was calibrated. The calibration model is the same as in Figure 3. During the calibration process, the rotational speed is set to zero, and all other settings remain unchanged in Table 1. In the calibration, the distance between the sensor and the blade is adjusted by changing the gap between them, and then the output voltage values of the sensor are obtained at different gaps to obtain the calibration curve of the sensor. Since all the blades are identical in size and material, a random blade from the disk was selected for calibration. First, to accurately obtain the coil voltage output and ensure alignment, the selected blade was adjusted to overlap with the coil’s centerline. The base coil voltage output was measured using a sinusoidal excitation signal with a frequency of 1 MHz and an amplitude of 3 V, as specified in Table 1. Next, the selected blade was moved into contact with the sensor (Tip clearance = 0). The distance between the blade and the sensor was then incrementally increased along the radial direction of the blade from 0 to 5 mm in steps of 200 μm. At each position, the sensor’s voltage output was recorded. The calibration results, shown in Figure 11, demonstrate the high sensitivity of the ECS to small distance variations. The maximum sensitivity reaches 7 mV/mm within the range of 1–2 mm, highlighting the ECS’s potential in BTC measurement. The calibration curve was fitted using a five-curve fitting formula, Equation (2), and resulting in a high fitting degree with R^2^ = 0.99997.

(2) $$V=a_{0}+a_{1}*d^{1}+a_{2}*d^{2}+a_{3}*d^{3}+a_{4}*d^{4}+a_{5}*d^{5}$$

wherein, $a_{0}=2940.67389$, $a_{1}=18.50394$, $a_{2}=-4.70861$, $a_{3}=0.65672$, $a_{4}=-0.03996$, $a_{5}=0.000152079$.

### 4.2. Rotation Speed

**Figure 12:**
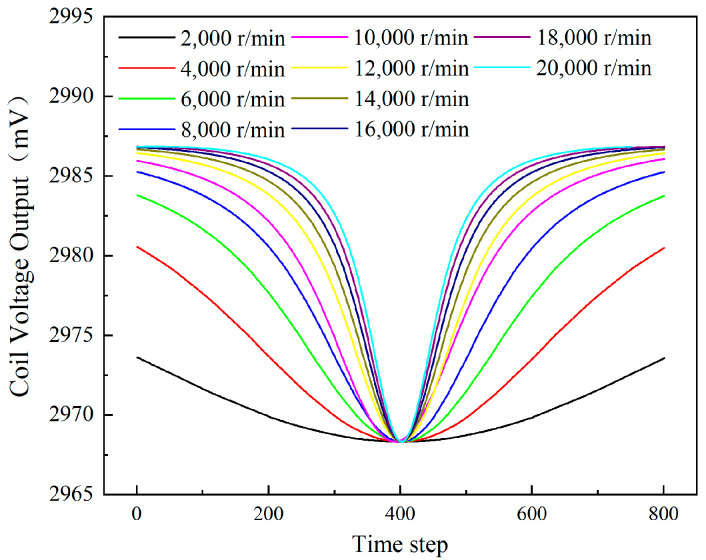
The sensor voltage output with time at different rotational speeds after shifting phase.

**Figure 13:**
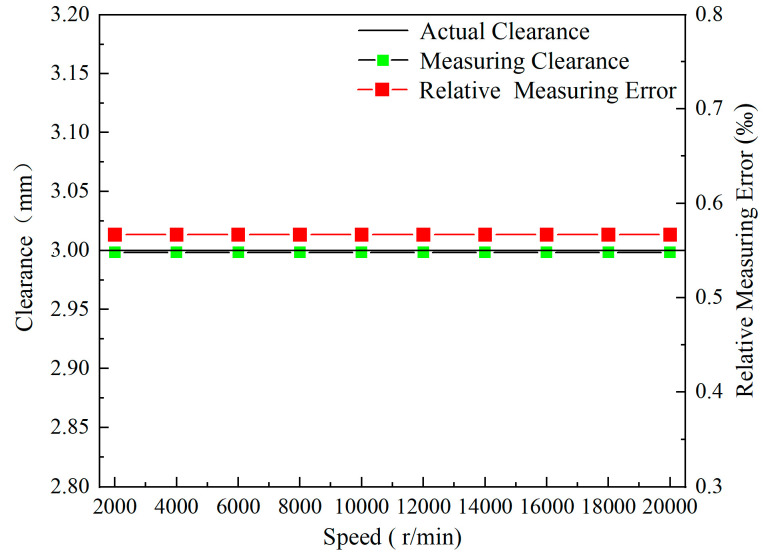
Measuring clearance and relative measuring error at different rotational speeds.

Under the condition of a 3 mm clearance, the influence of different rotation speeds on the coil voltage output was calculated. To visually compare the voltage output of the coil as the blades pass through the sensor, the voltage output curves were phase-shifted, as shown in Figure 12. The results indicate that the valley of the sensor voltage output remains constant when the speed ranges from 2000 r/min to 20,000 r/min. Figure 13 shows the measuring clearance values and relative measuring error, calculated based on the calibration curve (Figure 11) and the voltage values in Figure 12. The formula for calculating measuring relative error is shown in Equation (3).

(3) $$\delta =\frac{\left|d-d_{0}\right|}{d_{0}}\times 100$$

where *δ*—relative error; *d*—measuring clearance, mm; and *d_0_*—actual clearance, mm.

It can be seen that the measured clearance values remain stable and nearly match the actual values, with the relative measuring errors staying below 0.06%. Based on these results, all the subsequent investigations are conducted at a speed of 20,000 r/min to significantly reduce calculation time.

The influence of rotational speed on the eddy current sensor (ECS) predominantly affects the velocity of the blade tip. Drawing on insights from the literature, specifically reference [10], it is understood that eddy currents are induced as the rotating blade intersects with the magnetic induction lines generated. The density of these eddy currents is intimately linked to the magnetic flux variation rate, which, in turn, is dependent on the rotation speed of the blade. Despite rotational speeds reaching up to 20,000 r/min, resulting in a blade tip velocity of 157 m/s, this alteration in magnetic flux is considerably minor compared to the eddy currents induced by the sensor’s high-frequency excitation, which operates at a frequency of 1 MHz.

The high-frequency excitation of the sensor significantly overshadows the minimal variations in magnetic flux brought about by changes in rotational speed. Therefore, the influence of rotational speed on the variance of eddy currents is deemed negligible. Consequently, it is postulated that rotational speed does not impact the sensor voltage output. This understanding underscores the robustness of the ECS against variations in rotational speed, ensuring that its performance remains stable and accurate across a wide range of operational velocities.

**Figure 14:**
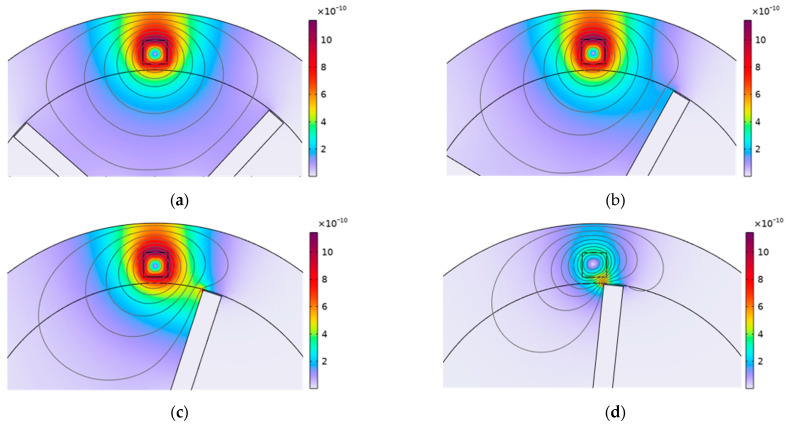
The distribution of magnetic flux density (T) for the same blade at different times, (**a**) t = 0.004 ms; (**b**) t = 0.004 ms; (**c**) t = 0.005 ms; (**d**) t = 0.006 ms.

The measured error can be interpreted through Figure 14, where the rotation speed is 2000 r/min. The figure illustrates the distribution of magnetic flux density for the same blade at various time intervals. As the blade penetrates the primary magnetic field area generated by the sensor, a secondary magnetic field begins to manifest on the surface of the blade tip. This secondary magnetic field arises due to the eddy currents induced by the changing primary magnetic field. As the blade continue to approach the sensor, the secondary magnetic field becomes stronger. According to Faraday’s law of electromagnetic induction, a decrease in secondary magnetic field intensity occurs around the blade tip, which, in turn, results in a decrease in sensor impedance. Consequently, the sensor voltage output also decreases. To mitigate this error, future calibration procedures may need to incorporate dynamic conditions that more accurately reflect the operational environment of the sensor. This would ensure that the sensor’s output is calibrated for the specific conditions under which it is used, thereby enhancing the accuracy and reliability of the measurements.

### 4.3. Blade Number

**Figure 15:**
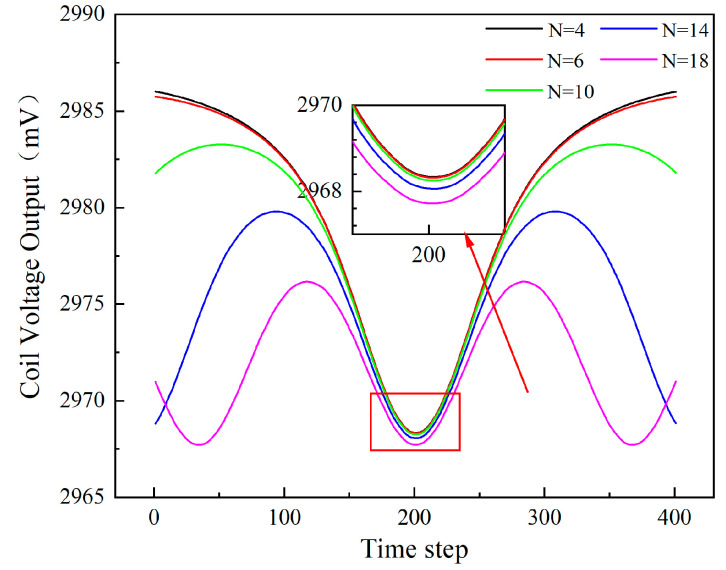
The sensor voltage output over time for different numbers of blades after shifting phase.

Figure 15 illustrates the relationship between the number of blades and the sensor voltage output. With an increase in the number of blades, there is a noticeable decrease in the minimum value of the sensor voltage. The data highlight that the presence of more blades results in lower valleys in the sensor output values, with the extent of reduction becoming progressively larger, as observed in the partially enlarged image of the figure.

**Figure 16:**
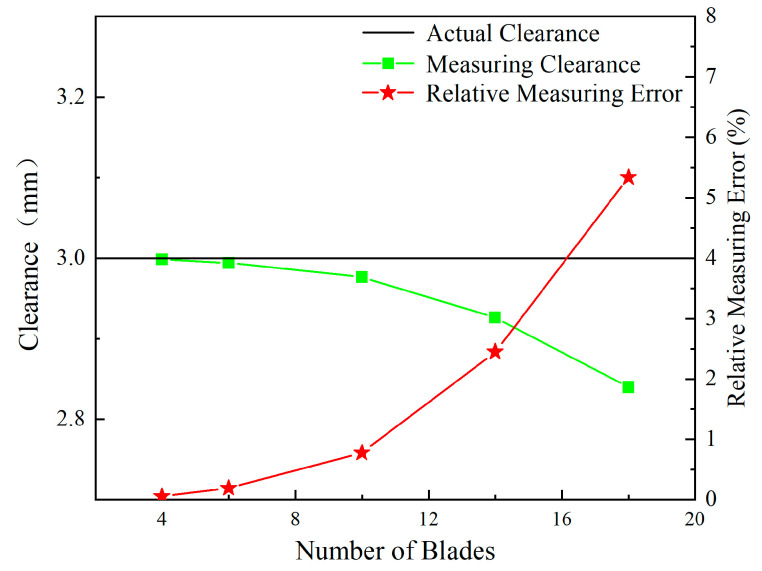
Measuring clearance and relative measuring error for different numbers of blades.

Following this observation, Figure 16 demonstrates a trend in the actual measured clearance values obtained by the sensor, which decrease as the number of blades increases. These measurements were calculated using the calibration curve presented in Section 3.5. Along with the decrease in clearance values, there is a gradual increase in the relative measurement error, which starts from nearly 0 and escalates up to 5.5%.

**Figure 17:**
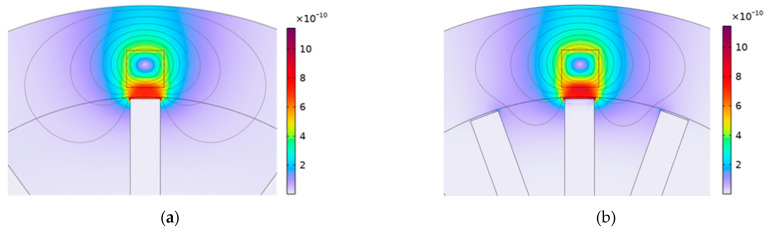
The distribution of magnetic flux density (T) for different numbers of blades when the blade passes through the sensor, (**a**) the number of blades is 10; (**b**) the number of blades is 18.

Figure 17 significantly aids in clarifying the impact of blade number on the operation of the eddy current sensor (ECS). This figure visually represents the magnetic flux density surrounding the blade tip when comparing configurations with 10 blades and 18 blades on the disk. Notably, the magnetic flux density appears more intensified when 18 blades are used. This increase can be attributed to the cumulative effect of magnetic fields from the closely spaced, adjacent blades, which enhances the overall magnetic field intensity around the blade tip.

According to Faraday’s law of electromagnetic induction, as the secondary magnetic field intensity around the blade tip strengthens, the impedance of the sensor decreases. When considering the electrical circuit shown in Figure 2 and applying the voltage divider rule, a noteworthy observation emerges: with an increase in the number of blades, the impedance of the sensor decreases. Consequently, this reduction in impedance results in a lower voltage across the sensor.

### 4.4. Blade Length

**Figure 18:**
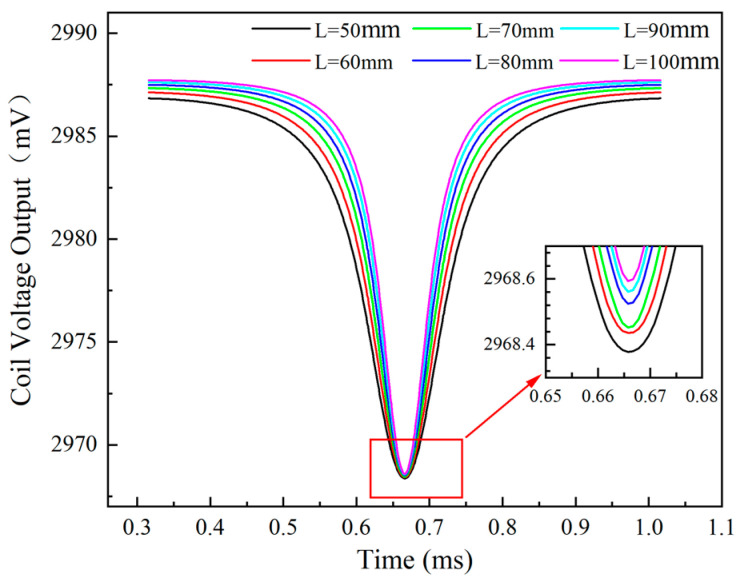
The sensor voltage output over time for different blade lengths.

Figure 18 shows the effect of the blade length, which ranges from 50 mm to 100 mm, on the voltage output of the ECS, achieved through phase shifting of the raw data. As the blade length increases, the valley of the sensor voltage output also increases.

**Figure 19:**
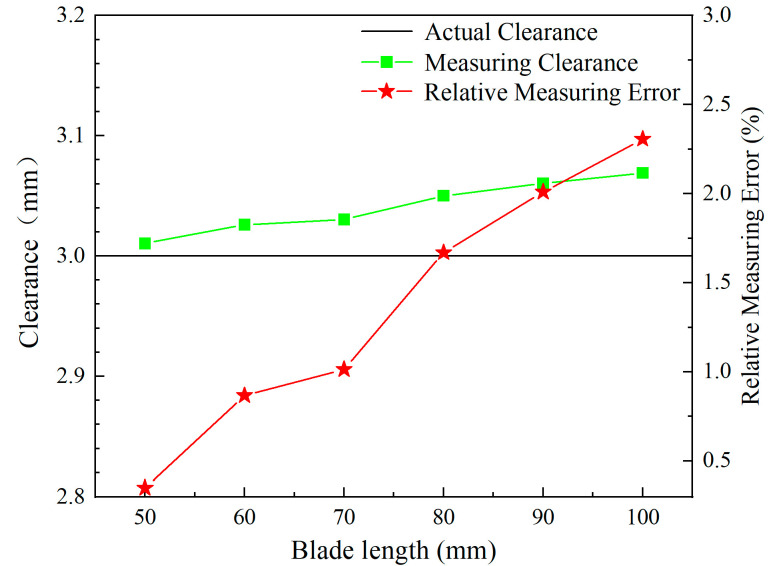
Measuring clearance and relative measuring error for different blade lengths.

In conjunction, Figure 19 indicates that the actual measured clearances of the sensor become progressively larger as the blade length increases, calculated using the calibration curve from Section 3.5. The maximum error approaches 50 μm, with the relative measurement error gradually increasing to 2.3%.

**Figure 20:**
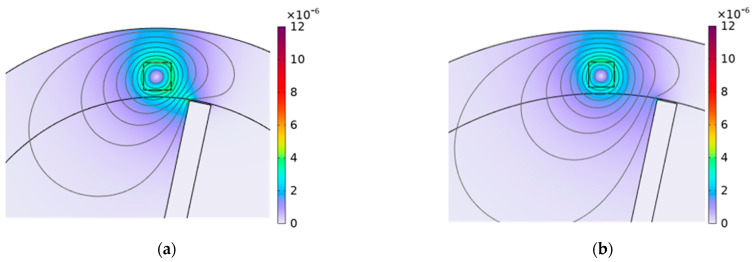
The distribution of magnetic flux density (T) for different blade lengths at t = 0.65 ms, (**a**) the blade length is 50 mm; (**b**) the blade length is 100 mm.

**Figure 21:**
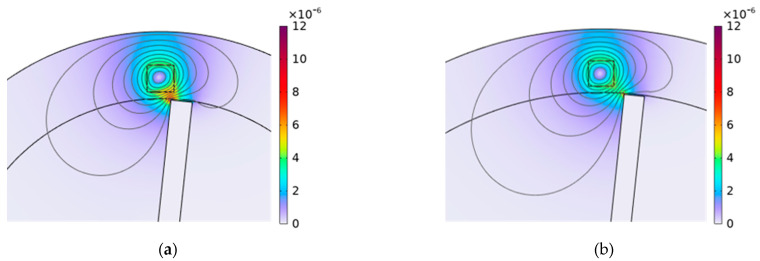
The distribution of magnetic flux density on the sensor (T) for different blade lengths at t = 0.7ms, (**a**) the blade length is 50 mm; (**b**) the blade length is 100 mm.

**Figure 22:**
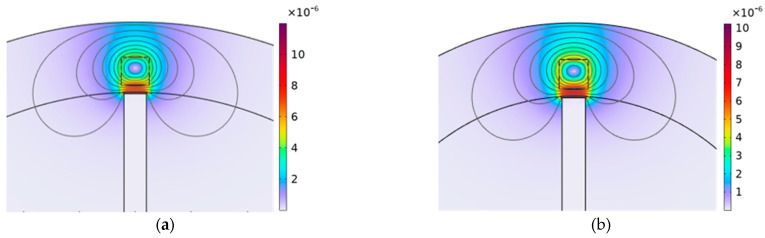
The distribution of magnetic flux density (T) for different blade lengths at t = 0.75 ms, (**a**) the blade length is 50 mm; (**b**) the blade length is 100 mm.

The effect of the blade length on ECSs can be further clarified by Figure 20, Figure 21 and Figure 22, which show the distribution of magnetic flux density around the sensor for both the shortest and longest blades at different times. From the figure, it can be observed that the magnetic field density at the short blades is stronger compared to that at the long blades at the same moment. Additionally, the figures show that the magnetic field density at the blades gradually increases over three different moments. As a result, when the blades pass through the sensor, the magnetic field density at the short blades is stronger than that at the long blades, as shown in Figure 22.

Ultimately, the magnetic flux density is more pronounced for the shortest blade than for the longest blade. Therefore, based on Faraday’s law of electromagnetic induction, a greater magnetic field intensity around the blade tip causes a reduction in impedance of the sensor. This relationship can be understood in the context of the electrical circuit depicted in Figure 2 and the voltage divider rule. As the blade length increases, the weak magnetic field around the blade tip results in greater impedance. Consequently, the sensor voltage increases with the blade length.

### 4.5. Radius of the Rotor Hub

**Figure 23:**
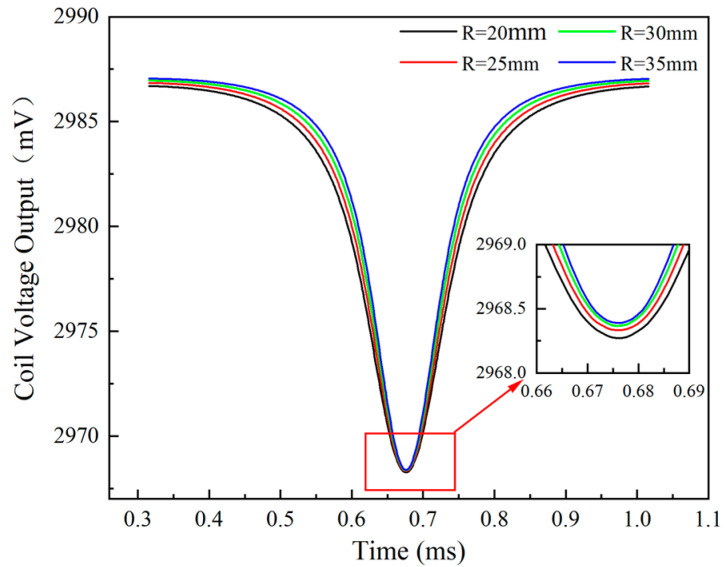
The sensor voltage output over time for different radii of disk.

Figure 23 illustrates the effect of varying the radius of the rotor hub on the sensor voltage output. Similar to the relationship observed with blade length, the sensor voltage output increases with an increase in either blade length or rotor hub radius. However, the minimum voltage growth rate gradually decelerates as the radius of the rotor hub increases.

**Figure 24:**
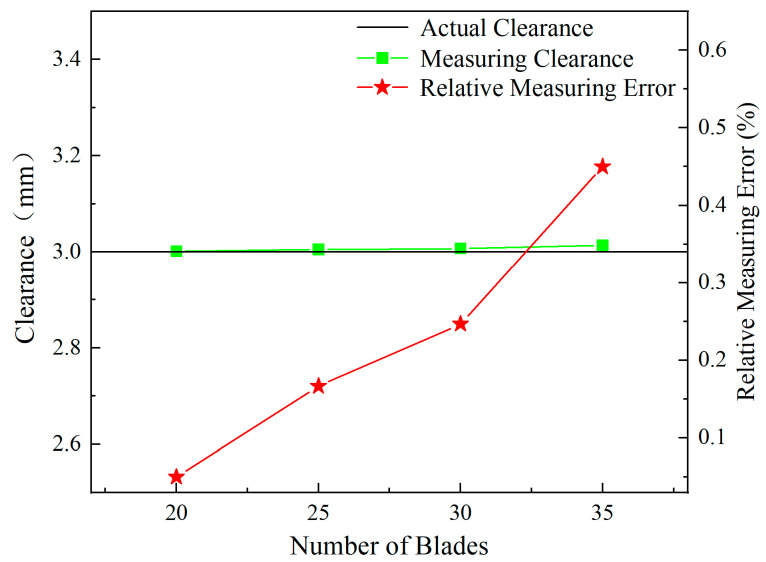
Measuring clearance and relative measuring error for different radii of rotor hub.

Figure 24, based on the sensor voltage data, presents the measuring clearance calculated using the calibration curve from Section 3.5. Both the measuring clearance and the relative measuring error increase with an increase in the radius of the rotor hub. Notably, the maximum relative measuring error is only 0.05%, and the overall maximum relative measuring error remains below 0.45%.

As an increase in the radius of the rotor hub is equivalent to an increase in the blade length, the effect of the radius on the eddy current sensor (ECS) can be analogized to the effect of blade length. As the radius increases, the intensified magnetic field around the sensor results in lower impedance. Consequently, the coil voltage decreases with the blade length.

## 5. Conclusions

In conclusion, this paper has successfully established a numerical computation model for blade tip clearance measurement using eddy current sensors (ECSs) and validated the model through experimental results. The simulations provided ECS voltage outputs that correspond to various turbine parameters. The parameter studies indicate that the ECS output is highly sensitive to certain geometric parameters. This sensitivity is crucial for accurately estimating potential measurement errors by considering these parameters. Furthermore, the research demonstrates that traditional ECS calibration methods introduce errors in the measurement of dynamic blade tip clearance (BTC). The detailed conclusions are as follows:

1. Rotation Speed Variation: The variation in rotation speed has no effect on the ECS voltage output. This finding implies that an ECS calibrated at a single speed can be reliably used for BTC measurements across different rotational speeds.
2. Geometric Parameter Sensitivity: Parameters such as blade number, blade length, and rotor hub radius significantly impact the ECS output. Notably, the trend in ECS voltage output changes differently with blade length or rotor hub radius compared to an increase in blade number. Specifically, while an increase in blade number raises the ECS voltage output, increases in blade length or rotor hub radius produce an opposite trend.
3. Calibration Under Dynamic Conditions: For accurate BTC measurement using ECS, it is essential to calibrate the sensor against the actual measurement target and under dynamic conditions. This approach helps establish a reliable baseline, thereby enhancing the precision of clearance measurements.

These detailed conclusions underscore the importance of accounting for specific geometric parameters and dynamic conditions in ECS calibration, thereby improving the accuracy and reliability of BTC measurements.

## Figures and Tables

**Table 1:** Specification of the blade disk configuration.

Name	Value	Unit	Description
R	25	mm	Radius of the rotor hub
L	50	mm	Blade length
b	8	mm	Blade width
w	10	mm	Sensor width
l	10	mm	Sensor length
Tip	3	mm	Gap between sensor and blade
Rs	R + L + 0.5 + 100	mm	Radius of static air area
Rr	R + L + 0.5	mm	Radius of rotating air area
n	13	turns	Number of turns in the coil
U	3	V	Voltage amplitude of the excitation
*f*	1	MHz	Frequency of the excitation
*fs*	20	MHz	Sampling frequency
*t*	1/*fs*	s	Time step
R0	10	Ω	Series resistance
N	4		Blade number

## Data Availability

The data presented in this study are available on request from the corresponding author.
